# Supplementary material for: Development and Initial Validation of the Duke Misophonia Questionnaire
Source: Front Psychol. 2021 Sep 29;12:709928. doi: 10.3389/fpsyg.2021.709928 (PMC8511674; doi:10.3389/fpsyg.2021.709928)
Supplement: Supplementary file 1 [file Table_1.DOCX]

Supplemental Table 1. All items generated for Duke Misophonia Questionnaire.

| **Items** |
| --- |
| **Affective Response Subscale** |
| I felt distressed. |
| I felt upset. |
| I felt guilty. |
| I felt scared. |
| **I felt hostile.** |
| I felt grouchy. |
| I felt ashamed. |
| I felt nervous. |
| **I felt jittery.** |
| I felt afraid. |
| **I felt anxious.** |
| I felt annoyed. |
| **I felt angry.** |
| **I felt disgusted.** |
| I felt unhappy. |
| **I felt frustrated.** |
| I felt rage. |
| I felt sad. |
| I felt disdain. |
| **I felt hateful.** |
| I felt embarrassed. |
| I felt devastated. |
| **I felt panic.** |
| I felt empathy for the person making the sounds. |
| I felt disengaged. |
| I felt detached. |
| I felt disconnected. |
| I felt empty. |
| I felt vacant. |
| My mind went blank. |
| I felt dazed. |
| **Physical Response Subscale** |
| I felt dizzy or lightheaded. |
| I had pain in my chest. |
| **I trembled or shuddered.** |
| **My heart pounded or raced.** |
| I felt nauseous. |
| I had an upset stomach. |
| I had trouble catching my breath. |
| I had hot spells. |
| I had cold spells. |
| I felt numbness or tingling in parts of my body. |
| I had a lump in my throat. |
| I felt physical pain. |
| I felt panicky. |
| I had goosebumps. |
| I felt my muscles tense up or tighten. |
| I started sweating. |
| I held my breath. |
| I had a headache. |
| I had groin sensitivity. |
| I clenched my teeth. |
| My ears hurt. |
| I flinched. |
| I felt like I was being prodded with a needle. |
| I felt a sensation similar to an electric shock. |
| I felt on edge. |
| I felt tension around my ears. |
| I had the urge to urinate. |
| My face got red. |
| **I became rigid or stiff.** |
| **I started breathing intensely or forcefully.** |
| I had a migraine. |
| I started tearing up. |
| **I reflexively jumped.** |
| **Cognitive Response Subscale** |
| **"I am helpless."** |
| "There is no hope." |
| "I resent the person making the sound." |
| "I wish that my senses would stop working." |
| "I am afraid I will hear that sound again." |
| "I want another person to know how upset I am." |
| "I want the person to stop making the sound." |
| "I want to force the person, thing or animal to stop making the sound." |
| "I want to mask the sound with something else." |
| "I want get physically far away from the sound." |
| "I am afraid I might hurt someone's feelings." |
| "I do not want to make a scene." |
| "I want to get away from the sound as quickly as possible, even if it would make a scene." |
| "I want to scream." |
| **"I want to cry."** |
| "I want to get revenge." |
| "I feel violated." |
| "This person is invading my space." |
| "They do not need to keep making this sound." |
| "Don't they know they are making this awful sound?" |
| "Don't they know how often they are making this awful sound?" |
| "Why can't they stop making this sound." |
| "They did not learn manners." |
| "They should not be making this sound." |
| "They do not care how this sound affects me." |
| "They know this sound hurts me and are making it on purpose." |
| "They are being disrespectful." |
| "They should stop making this sound." |
| "I am being irrational." |
| "Something is wrong with me." |
| "I am not normal." |
| "This is my fault." |
| "I hate the person making the sound." |
| "I am worried I might hurt the person making the sound." |
| **"How do I make this sound stop?"** |
| "Stop it!" |
| "I wish I could stop paying attention to the sound." |
| **"I need to get away from the sound."** |
| **"I would do anything to make it stop."** |
| "I hate this sound." |
| "Oh no! It's happening again!" |
| "Where is this sound coming from?" |
| "Why is this happening?" |
| "I would rather be dead than hear this." |
| "Who is making this sound?" |
| "They are going to do it again." |
| **"Everything is awful."** |
| "How will I live through this sound?" |
| "I can't escape." |
| "This is horrible." |
| "I can't believe they are doing this." |
| "I wish I could get over it." |
| "Why am I like this?" |
| "I am being attacked." |
| "I have to leave." |
| "How will I live my life like this?" |
| "They should know better." |
| "I need to make the sound stop." |
| "I should not be acting like this." |
| **"I cannot handle this."** |
| "This is not fair." |
| "This person is disgusting." |
| "Why can't they eat normally?" |
| **I thought about pushing, poking, shoving etc. the person making the sound.** |
| **I thought about screaming at, yelling at or telling off the person making the sound.** |
| **I thought about physically hurting the person making the sound.** |
| I imagined being violent toward the person making the sound but would never act on it. |
| **Behavioral Response Subscale** |
| I physically hurt someone. |
| I yelled at, screamed at, or told someone off. |
| I held myself back from physically hurting someone. |
| I held myself back from yelling at, screaming at, or telling someone off. |
| I nicely asked the person to stop making the sound. |
| I ordered the person to stop making the sound. |
| I discreetly covered one or both ears. |
| I calmly moved away from the noise. |
| I overtly covered my ears. |
| I mimicked the person making the sound. |
| I made repetitive sounds. |
| I turned away so that I did not see the person making the sound. |
| I pushed, poked, shoved etc. the person making the sound. |
| I immediately left the room to escape the sound. |
| I screamed. |
| I cried. |
| I physically hurt an animal. |
| I looked or stared at the person making the sound. |
| I showed my distress using my facial expression and/or body language (e.g., squinting my eyes, crossing my eyes, sighing loudly). |
| I blinked more. |
| I indirectly attempted to stop the sound (e.g., hand the person a tissue to get them to stop sniffling). |
| I took the sound producing object away from the person. |
| I kept asking the person to stop until they did. |
| I moved away from the sound. |
| I explained that the sound is bothersome to me. |
| I looked around for the sound source. |
| I purposefully tensed my muscles. |
| I held my breath. |
| I used ear plugs or headphones. |
| I rubbed my head. |
| I pulled my hair. |
| I tried to pop my ears. |
| I pushed one foot into the other. |
| I caused myself pain (e.g., dug my nails into my skin, bit my lip, pushed an object into my skin). |
| I showed how angry I was by acting aggressively (e.g., broke a pencil, slammed things). |
| I closed my eyes. |
| I blocked the sound source from my view (e.g., covered face with hair, covered eyes with hands, moved head so something else was in the way). |
| I made jerky movements. |
| I ran out of the room. |
| I produced an alternate sound (e.g., humming). |
| I grabbed my groin. |
| I clenched my fists. |
| I made repetitive movements (e.g., tapping, pacing). |
| **Coping-Before Subscale** |
| **I avoided certain people, places, or things so I would not have to hear sounds I dislike.** |
| I asked the person not to make a sound before they made it. |
| I did things to prevent a sound from occurring (e.g., asked person to take allergy pills, instituted a "no gum" policy). |
| I made it easy to escape if I needed to (e.g., sat near the door). |
| I positioned myself to limit sounds (e.g., moved computers away from my chair, sat facing away from a coworker). |
| I carried ear plugs, headphones or turned on ambient noise to block any sound that might occur. |
| I ate alone to avoid hearing bothersome sounds. |
| **I used a different sound to drown the bothersome sound (e.g., turned on TV).** |
| I determined where to live or work in order to avoid bothersome sounds. |
| **I used strategies to make myself less bothered by sounds I might hear (e.g., deep breathing, meditation, visualization).** |
| **I was on guard for bothersome sounds.** |
| I avoided looking at people or objects that might make a sound I dislike. |
| **I distracted myself so as not to be bothered by a sound I might hear.** |
| I held myself back from saying anything about a bothersome sound that might occur. |
| **I made a plan to cope with bothersome sounds if they occurred.** |
| I prepared myself to ignore the sound. |
| I isolated myself to avoid hearing a sound. |
| I described my sound sensitivity to others so they would understand. |
| I spent time learning about an event or activity to figure out if I would be bothered by the sounds there. |
| I brought soothing objects with me that would help me tolerate distress (e.g., soothing lotion, pictures). |
| I took medication to help me feel calmer. |
| I exercised to help me cope better. |
| I interacted with friends. |
| **Coping-During** **Subscale** |
| I asked the person to stop making the sound. |
| I left the room or situation to escape the sound. |
| I made another noise to cover the bothersome sound. |
| I posted on social media (e.g., Facebook) or an online community (e.g., Reddit) about the experience. |
| I asked for a break from the activity that put me in contact with the sound. |
| I alerted the person to the fact that they were making the sound. |
| I mimicked the person making the sound. |
| I used my facial expressions or body language to convey that I wanted the sound to stop (e.g., gave a side eye). |
| I texted or called someone else to help me cope with the ongoing sound. |
| I forced myself to tolerate the sound, i.e., "white knuckled it." |
| I acted like I was not bothered by the sound. |
| **I blocked the sound (e.g., covered ears with hands, headphones, ear plugs).** |
| **I increased the background noise to cover up the bothersome sound (e.g., turned on TV, rolled down car window).** |
| I moved to a different position. |
| I created a different physical sensation (e.g., dug nails into palm). |
| **I used strategies to calm myself (e.g., self-talk, breathing exercises).** |
| I deliberately focused my attention on the sound. |
| I tried to suppress thoughts about the sound. |
| **I changed my way of thinking about the sound.** |
| I took my anger out on an object. |
| I tried to soothe myself (e.g., ate comforting food, used a weighted blanket). |
| **I reminded myself that it could be worse.** |
| I removed the source of the sound. |
| **I looked away from the source of the sound.** |
| I indirectly asked the person to stop the sound (e.g., traded pens for one that does not click). |
| I asked a family member or friend to talk to others about the sound on my behalf. |
| I explained to the person that I have a condition that makes me sensitive to certain sounds. |
| I chewed at the same rate as the other person. |
| I reminded myself that the sound will end. |
| **I focused my attention on an activity (e.g., watched TV or videos).** |
| **I mindfully focused on current sensations without judgment.** |
| **I listened to music or a different sound.** |
| **I produced an alternate sound (e.g., humming).** |
| **Coping-After Subscale** |
| I told the person not to make the sound in the future. |
| I thought about what caused the sound. |
| I turned to others for emotional support. |
| I was nicer to the person making the sound than I had been before. |
| I told the person the effect the sound had on me. |
| I apologized for my reaction to the sound. |
| I told the other person about my sound sensitivity. |
| **I thought about strategies to help me cope better next time.** |
| I isolated myself from sources of unpleasant sounds. |
| **I did something to comfort myself (e.g., exercised, went somewhere calming, pet animals).** |
| I focused my attention on something else (e.g., watched TV, called a friend). |
| **I listened to a comforting sound (e.g., white noise, music).** |
| **I used the sight, smell or touch of an object to soothe myself (e.g., looked at a soothing picture, smelled a scent, or touched a soft blanket).** |
| I grounded myself in the present moment. |
| I laid down. |
| I sought out validation from social media (e.g., Facebook) or an online community (e.g., Reddit). |
| I held myself back from saying anything. |
| I wrote about my experience in a journal, blog, etc. |
| I asked the person why they made the sound. |
| I asked a family member or friend to talk to others on my behalf about the sound(s). |
| I discussed my concerns with a therapist or professional. |
| I went to sleep. |
| I suppressed thoughts about what happened. |
| I re-focused my attention on what I was doing before the sound occurred. |
| **I did some relaxation exercises (e.g., deep breathing, meditation).** |
| **Impairment Subscale** |
| My physical or mental health |
| My ability to interact in a group |
| **My ability to be with other people** |
| **My ability to function in daily activities without help** |
| How much time I spend with my family |
| The quality of my family relationships |
| Whether I feel like a good role model |
| **How much I enjoy spending time with my family** |
| What kind of future I imagine for my family |
| What kind of future I imagine for my long-term relationships |
| What I think about myself |
| **My self-esteem** |
| My confidence |
| How much I think other people like me |
| **How connected I feel to other people** |
| My productivity at work or school |
| **My performance at work or school** |
| **My ability to maintain employment** |
| My ability to gain employment |
| **My ability to work with others** |
| My ability to work without accommodations |
| My sleep |
| My day-to-day activities (e.g., meals, bathing, chores) |
| **The quality of my romantic relationships** |
| My desire to have a partner |
| My desire to seek out a partner |
| How much time I spend with my romantic partner |
| My ability to stay in a romantic relationship |
| How much time I spend with friends |
| What activities I'll do with friends |
| **The quality of relationships with my friends** |
| My ability to make new friends |
| My ability to keep friends |
| The kinds of activities I do with my family |
| The kinds of activities I do with my romantic partner |
| My finances (due to expenses related to my condition) |
| The relationships my family members have with each other (not including me) |
| **My ability to "be myself"** |
| My attendance at work or school |
| The kinds of people I am friends with |
| The number of friends that I have |
| **My ability to live with other people (e.g., roommate, partner)** |
| The kinds of activities I do by myself |
| **Beliefs Subscale** |
| "No one understands my problems with certain sounds." |
| "My sound issues do not seem to have a known cause." |
| "I am isolated." |
| "Others think I am crazy." |
| "No one can help me with my sound issues." |
| **"My sound issues will only get worse with time."** |
| "My sound issues have not been recognized as legitimate." |
| **"My whole life will be affected by sound issues."** |
| "I am not normal." |
| "It is difficult to explain what I am going through." |
| **"People do not understand me."** |
| "Nobody loves me." |
| "I am not a whole person." |
| "Nobody will ever love me." |
| **"I am a burden on others."** |
| "No one believes me." |
| **"No one can help me."** |
| **"I hate being like this."** |
| "I am being too difficult." |
| "No one cares about me." |
| "I am not like others." |
| "People do not understand my experience." |
| "Others are inconsiderate." |
| **"This is unfair."** |
| "I am missing out on a lot." |
| "I am a bad person." |
| "People think I am crazy." |
| **"I am crazy."** |
| "I am not happy." |
| "This consumes my attention." |
| **"I should get over it."** |
| "This is ridiculous." |
| **"I should have known how to cope earlier."** |
| "I have suffered." |
| "This will affect my long-term relationships." |
| **"I am weak."** |
| "I have a disability." |
| "I need to work harder than others to make up for this problem." |
| "I need to control this experience." |
| **I should be able to control my reaction to these sounds** |
| **"My reactions to sounds are irrational."** |
| **"I will be rejected if people find out."** |
| "I am a hypocrite as I make similar sounds myself." |
